# Hypercholesterolemia Impairs the Expression of Angiogenic MicroRNAs in Extracellular Vesicles Within Ischemic Skeletal Muscles

**DOI:** 10.3390/ncrna12010003

**Published:** 2026-01-26

**Authors:** Nozha Raguema, Sylvie Dussault, Kevin Sawaya, Michel Desjarlais, Eric Boilard, Sylvain Chemtob, Alain Rivard

**Affiliations:** 1 Department of Medicine, Centre Hospitalier de l’Université de Montréal (CHUM) Research Center, Montreal, QC H2X 0A9, Canada; nozha.raguema@umontreal.ca (N.R.); sylvie.dussault.chum@ssss.gouv.qc.ca (S.D.); kevin.sawaya@umontreal.ca (K.S.); 2 Departments of Pediatrics, Ophthalmology and Pharmacology, Centre Hospitalier Universitaire Sainte-Justine Research Center, Montreal, QC H3T 1C5, Canada; michel.desjarlais@umontreal.ca (M.D.); sylvain.chemtob@umontreal.ca (S.C.); 3 Department of Infectious Diseases and Immunity, Centre de Recherche du Centre Hospitalier Universitaire de Québec, Université Laval, Quebec, QC G1V 4G2, Canada; eric.boilard@crchudequebec.ulaval.ca

**Keywords:** hypercholesterolemia, extracellular vesicles, microRNA (miR), ischemia, angiogenesis, neovascularization

## Abstract

**Background/Objectives**: In severe peripheral artery disease (PAD) with limb ischemia, hypercholesterolemia (HC) is associated with impaired neovascularization. Extracellular vesicles (EVs) are present within ischemic muscles, and they contain microRNAs (miRs) involved in several biological functions, including angiogenesis and neovascularization. **Methods**: We used a mouse model of PAD and compared the response to hindlimb ischemia in hypercholesterolemic ApoE*^−/−^* vs. normocholesterolemic mice. Next-generation sequencing (NGS) was used to perform full miR expression profiling in ischemic skeletal muscles and in EVs of varying sizes—large EVs (lEVs) and small EVs (sEVs)—within these muscles. **Results**: We identified several miRs with potential pro-angiogenic effects (angiomiRs) that are reduced by HC in lEVs (Let-7b-5p, miR-151-3p, Let-7c-5p) or sEVs (miR-21a-5p, miR-196b-5p, miR-340-5p). As proof of principle, we showed that the overexpression of Let-7b-5p in lEVs, or miR-21a-5p in sEVs, can significantly increase the angiogenic capacity of these EVs in vitro. HC also impaired the enrichment of specific angiomiRs in lEVs (miR-100-5p), sEVs (miR-142a-3p), or in both lEVs and sEVs (miR-146b-5p). In silico approaches, including the prediction of miR targets, pathway unions, and gene unions, identified the resulting predictive effects of HC-modulated miRs in EVs on processes with key roles in the modulation of angiogenesis and neovascularization, such as the regulation of the actin cytoskeleton and focal adhesion and the HIF-1, MAPK, AMPK, and PI3K-Akt signaling pathways. **Conclusions**: Our results constitute an important first step towards the identification of specific miRs that could be targeted to improve EV angiogenic function in hypercholesterolemic conditions and reduce tissue ischemia in patients with severe PAD.

## 1. Introduction

Peripheral arterial disease (PAD) is a progressive condition in which the arteries, particularly those supplying blood to the limbs, become narrowed and hardened due to the buildup of atherosclerotic plaques [1]. This results in reduced blood flow and oxygen delivery to tissues, leading to ischemia. PAD is linked to exercise-induced skeletal muscle pain (claudication) and reduced mobility. In advanced stages, persistent ischemia can cause rest pain, non-healing ulcers, tissue necrosis, and gangrene, ultimately resulting in lower-limb amputation. In many patients, the atherosclerotic burden is so extensive that revascularization through angioplasty or bypass surgery is not feasible. Thus, boosting the body’s intrinsic ability to form new blood vessels (neovascularization) represents a promising approach to enhancing the perfusion of affected muscles, alleviating pain, and preventing ischemic tissue injury and limb loss [2].

Neovascularization is triggered by ischemic stimuli and encompasses both the growth of collateral arteries (arteriogenesis) and the activation, proliferation, and migration of endothelial cells (ECs) that expand the existing vascular network (angiogenesis) [3]. Angiogenesis is modulated by the surrounding microenvironment and various growth factors. Vascular Endothelial Growth Factor (VEGF) is a key driver of angiogenic initiation [4]. Under hypoxic conditions, VEGF expression is upregulated via hypoxia-inducible factor-1 (HIF-1) and acts on ECs to promote vascular permeability, EC proliferation, migration, and tubule formation [5]. VEGF signaling involves the activation of the p44/42 MAPK and p38 MAPK pathways, which support EC proliferation and migration, as well as PI3-kinase/Akt/NO signaling, which contributes to EC survival and motility [6,7]. It has been demonstrated that postnatal neovascularization also depends on the action of bone marrow-derived pro-angiogenic cells (PACs) [8,9] that can reach sites of ischemia and promote neovascularization through the paracrine secretion of angiogenic factors and cytokines [10].

Hypercholesterolemia (HC) is a major risk factor for the development of cardiovascular diseases, including PAD. In addition to accelerating the progression of atherosclerosis, HC also impairs the body’s ability to adequately respond to ischemia. In fact, HC has been shown to be associated with impaired blood flow recuperation and angiogenic response in several animal models [11,12,13]. Even in the absence of clinically apparent atherosclerotic diseases, HC by itself has also been shown to reduce the number and function of PACs in patients [14]. However, the precise mechanisms responsible for the detrimental effects of HC on vascular function, angiogenesis, and neovascularization are still largely unknown.

There is growing evidence that microRNAs (miRNAs or miRs) play an important role in the physiological response to ischemia. miRs are a class of endogenous, non-coding small RNAs (20–25 nucleotides) that repress protein synthesis by blocking mRNA translation or promoting mRNA degradation, thereby silencing gene expression. They modulate numerous physiological and pathological processes, including angiogenesis and neovascularization [15,16,17]. Consequently, enhancing the expression of pro-angiogenic miRs (angiomiRs) has been proposed as a potential therapeutic approach to augmenting ischemia-induced neovascularization in atherosclerotic disease [18,19,20]. miRs can be transferred between cells in a paracrine fashion through extracellular vesicles (EVs). Cells release various types of extracellular vesicles (EVs), including small EVs (sEVs, 30–200 nm) and large EVs (lEVs, 200–1000 nm), which serve as key mediators of intercellular communication under both physiological and pathological conditions [21,22,23]. The miR cargo of EVs, particularly the delivery of angiomiRs to recipient cells, appears to play a crucial role in promoting angiogenic responses [24]. Yet, in ischemic vascular diseases, the specific miRs incorporated into EVs within skeletal muscle—and how this packaging is influenced by HC—remain largely unclear. Using a mouse model of PAD, we employed next-generation sequencing (NGS) to comprehensively profile miR expression and assess the impact of HC in skeletal muscle and in EVs of different sizes (lEVs and sEVs) derived from ischemic tissue. Our findings show that multiple miRs with potential pro-angiogenic activity are reduced by HC in both lEVs and sEVs. Overall, these angiomiRs are predicted to regulate several major pathways that govern angiogenesis and neovascularization, highlighting their potential as therapeutic targets to enhance the angiogenic capacity of EVs and support neovascularization in PAD patients with critical limb ischemia.

## 2. Results

### 2.1. miR Expression Profile in Whole Skeletal Muscles and Modulating Effect of Hypercholesterolemia

**Figure 1:**
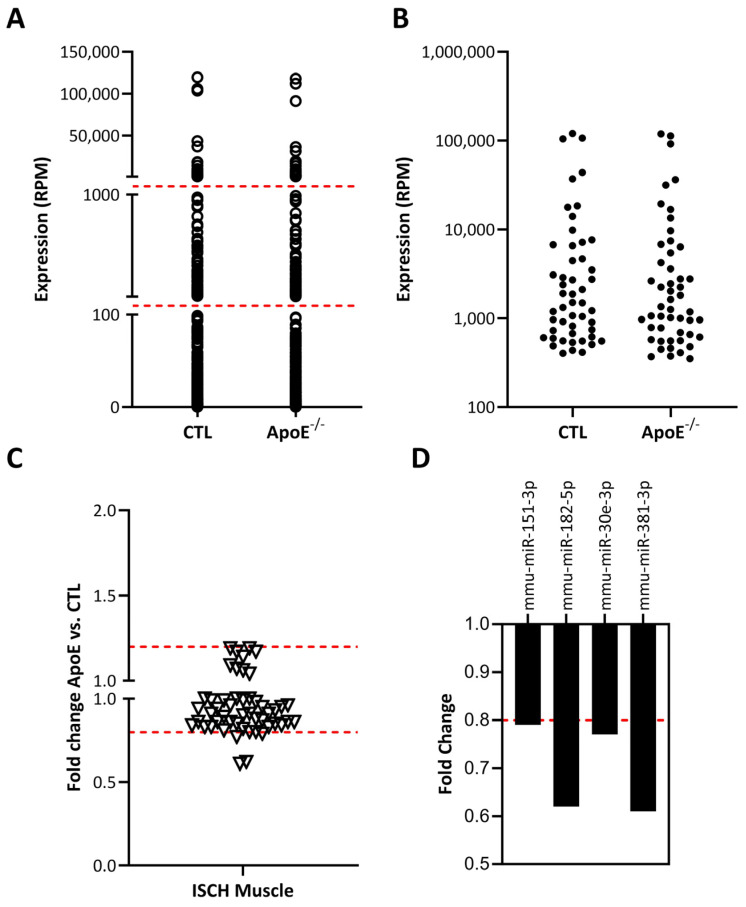
Effect of hypercholesterolemia on miR expression profile in whole ischemic skeletal muscles. (**A**,**B**). Expression profile of all miRs (**A**) or 50 most expressed miRs (**B**) isolated in whole ischemic skeletal muscles of hypercholesterolemic ApoE^−/−^ mice (pool of 3) or normocholesterolemic control mice (CTL, pool of 4). (**C**). Fold change representation of effect of hypercholesterolemia on 50 most expressed miRs in ischemic muscles. (**D**). Representation of 4 miRs that were reduced by more than 20% in hypercholesterolemic conditions, including 1 potential angiomiR (miR-151-3p).

Next-generation sequencing (NGS) was used to perform full miR expression profiling in skeletal muscles after the induction of hindlimb ischemia, and we compared the results in hypercholesterolemic ApoE^−/−^ mice vs. control normocholesterolemic mice. A total of 453 different miRs were detected in ischemic skeletal muscles (Figure 1).

The general distribution of miR expression in ischemic muscles was similar in hypercholesterolemic vs. normocholesterolemic mice (Figure 1A). Several miRs were found to have very low expression levels (i.e., <100 RPM), and therefore their physiological impact might be limited. Focusing on the 50 most expressed miRs in ischemic muscles, only a few candidates were shown to be modulated (mostly downregulated) by hypercholesterolemia (Figure 1B,C). Four miRs were shown to be reduced by at least 20% in the ischemic muscles of hypercholesterolemic ApoE ko mice (Figure 1D). Interestingly, among these miRs, miR-151-3p was previously found to promote angiogenesis in vitro [25], although its role in the modulation of angiogenesis and neovascularization in vivo is currently unknown.

### 2.2. Effect of Hypercholesterolemia on Ischemic Skeletal Muscle Large EV miR Cargo

**Figure 2:**
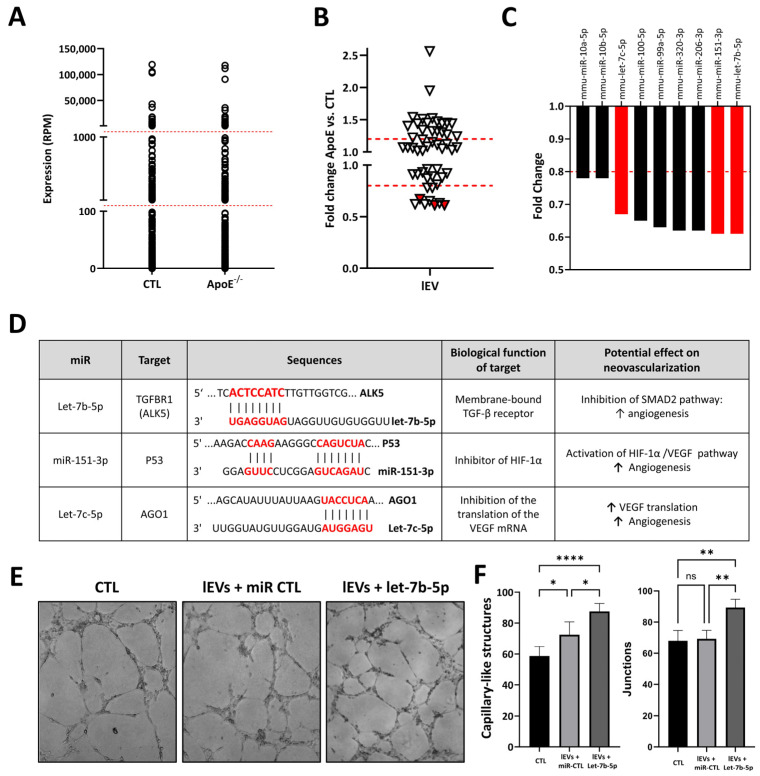
miR profile and effect of hypercholesterolemia in large extracellular vesicles (lEVs) isolated from ischemic skeletal muscles. (**A**). Expression profile of miRs in lEVs isolated from ischemic skeletal muscles of hypercholesterolemic ApoE^−/−^ mice (pool of 3) or normocholesterolemic control mice (CTL, pool of 3). (**B**). Fold change representation of effect of hypercholesterolemia on 50 most expressed miRs in lEVs isolated from ischemic skeletal muscles of hypercholesterolemic ApoE^−/−^ mice or normocholesterolemic control mice (CTL). (**C**). Representation of miRs in lEVs that are reduced by more than 20% in hypercholesterolemic conditions, including 3 potential angiomiRs shown in red. (**D**). Description of targets, biological function, and angiogenic effects of angiomiRs that are reduced by hypercholesteremia in lEVs. Binding sequences in target genes are shown in red. (**E**,**F**). Evaluation of activity of Let-7b-5p within lEVs in Matrigel capillary-like structure formation assay. lEVs were transfected with Let-7b-5p mimic or miR mimic control (miR CTL) before treatment of HUVECs. Number of capillary-like structures and junctions was determined. n = 5/group. ns = non significant * *p*˂0.05. ** *p* < 0.01. **** *p*˂0.001.

miRs can be transferred between cells by extracellular vesicles (EVs), including large EVs (lEVs) and small EVs (sEVs), and these vesicles have the ability to act as paracrine mediators in tissues. We performed a complete profiling of the miR cargo of lEVs and sEVs within the ischemic skeletal muscles of hypercholesterolemic vs. normocholesterolemic mice. In lEVs, 340 different miRs were detected. As shown in Figure 2A, the overall distribution of miRs is similar in lEVs within the ischemic muscles of hypercholesterolemic vs. normocholesterolemic mice, with several miRs exhibiting very low expression levels (i.e., <100 RPM) in both conditions. We focused on the 50 most highly expressed miRs in lEVs and found that hypercholesterolemia significantly altered the expression levels of several miRs (Figure 2B). Notably, nine miRs were downregulated by more than 20% under hypercholesterolemic conditions (Figure 2C). Among these, three were identified as potential angiomiRs within lEVs (Figure 2D). miR-151-3p, which was also identified in whole ischemic muscles, can improve angiogenesis through the activation of the HIF-1α/VEGF pathway [25]. Let-7b-5p is highly expressed in lEVs, and it has been shown to promote angiogenesis in vitro and in vivo through the targeting of anti-angiogenic TGFBR1 [26]. miR-let-7c is another member of the Let-7 family, which has been shown to target AGO1 [27], increase VEGF expression, and promote angiogenesis in vitro [28]. As proof of principle, we transfected lEVs with a Let-7b-5p mimic and found that the capacity to form capillary-like structures in a Matrigel assay is significantly increased in these engineered lEVs compared to native lEVs (Figure 2E,F). These findings are consistent with the pro-angiogenic role of Let-7b-5p within lEVs and suggest that a reduction in its expression could contribute to the impairment of lEV angiogenic activity and neovascularization in hypercholesterolemic conditions.

### 2.3. Effect of Hypercholesterolemia on Ischemic Skeletal Muscle Small EV miR Cargo

**Figure 3:**
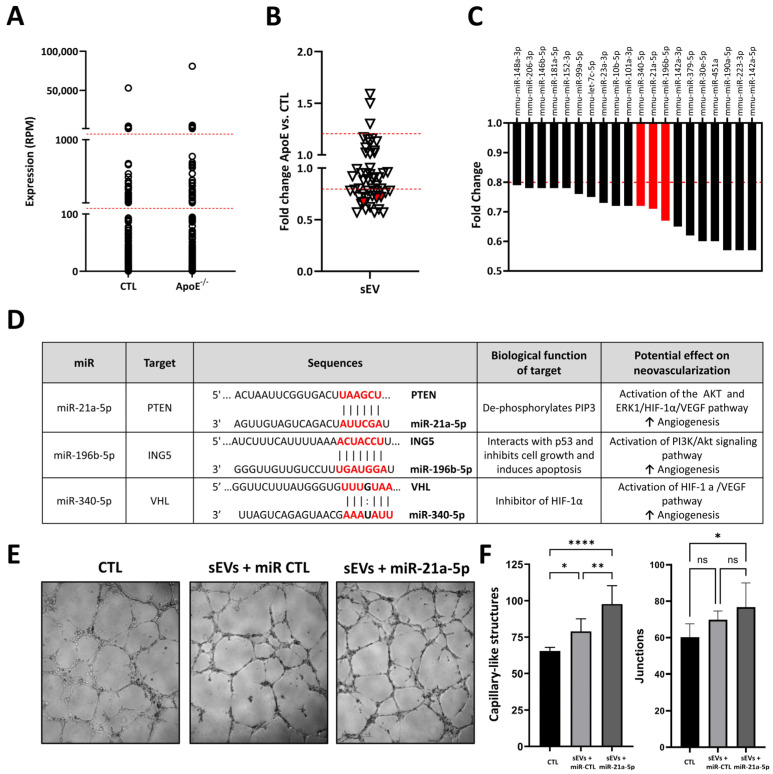
miR profile and effect of hypercholesterolemia in small extracellular vesicles (sEVs) isolated from ischemic skeletal muscles. (**A**). Expression profile of miRs in sEVs isolated from ischemic skeletal muscles of hypercholesterolemic ApoE^−/−^ mice (pool of 5) or normocholesterolemic control mice (CTL, pool of 4). (**B**). Fold change representation of effect of hypercholesterolemia on 50 most expressed miRs in sEVs isolated from ischemic skeletal muscles of hypercholesterolemic ApoE^−/−^ mice or normocholesterolemic control mice (CTL). (**C**). Representation of miRs in sEVs that were reduced by more than 20% in hypercholesterolemic conditions, including 3 potential angiomiRs shown in red. (**D**). Description of targets, biological function, and angiogenic effects of angiomiRs that are reduced by hypercholesteremia in sEVs. Binding sequences in target genes are shown in red. (**E**,**F**). Evaluation of activity of miR-21a-5p within sEVs in Matrigel capillary-like structure formation assay. sEVs were transfected with miR-21a-5p mimic or miR mimic control (miR CTL) before treatment of HUVECs. Number of capillary-like structures and junctions was determined. n = 8/group. ns = non significant * *p* < 0.05. ** *p* < 0.01. **** *p* ˂ 0.001.

A total of 236 different miRs were identified in sEVs within ischemic muscles. As shown in Figure 3A, the overall distribution of miRs is similar in sEVs isolated from the ischemic muscles of hypercholesterolemic vs. normocholesterolemic mice. As several miRs exhibited very low expression levels (i.e., <100 RPM), we focused on the 50 most highly expressed miRs in sEVs and found that hypercholesterolemia leads to significant modulation in the expression level of various miRs (Figure 3B). Notably, 20 miRs were shown to be reduced by more than 20% by hypercholesterolemia (Figure 3C). Among these, three were identified as potential angiomiRs within sEVs (Figure 3D). miR-21a-5p helps to preserve PIP3 phosphorylation and the activation of the AKT/mTOR/HIF-1α angiogenic pathway, through the targeting of PTEN [29]. miR-196b-5p targets ING5, which can activate the pro-angiogenic PI3K/Akt signaling pathway. Moreover, sEV-derived miR-196b-5p has previously been shown to reduce CDKN1B and increase angiogenesis in vitro [30]. miR-340-5p targets von Hippel–Lindau (VHL) [31], which can lead to the activation of the HIF-1a/VEGF pathway. Interestingly, serum sEV-derived miR-340-5p has previously been shown to promote angiogenesis in brain microvascular endothelial cells [32]. miR-21a-5p was the most highly expressed miRNA in sEVs. As proof of principle, we transfected sEVs with a miR-21a-5p mimic and found that the capacity to form capillary-like structures in a Matrigel assay is significantly increased in these engineered sEVs compared to native sEVs (Figure 3E,F). These findings are consistent with the pro-angiogenic role of miR-21a-5p within sEVs and suggest that a reduction in its expression could contribute to the impairment of sEV angiogenic activity and neovascularization in hypercholesterolemic conditions.

### 2.4. Hierarchical Ranking of miR Expression in Whole Ischemic Muscles, lEVs, and sEVs

**Figure 4:**
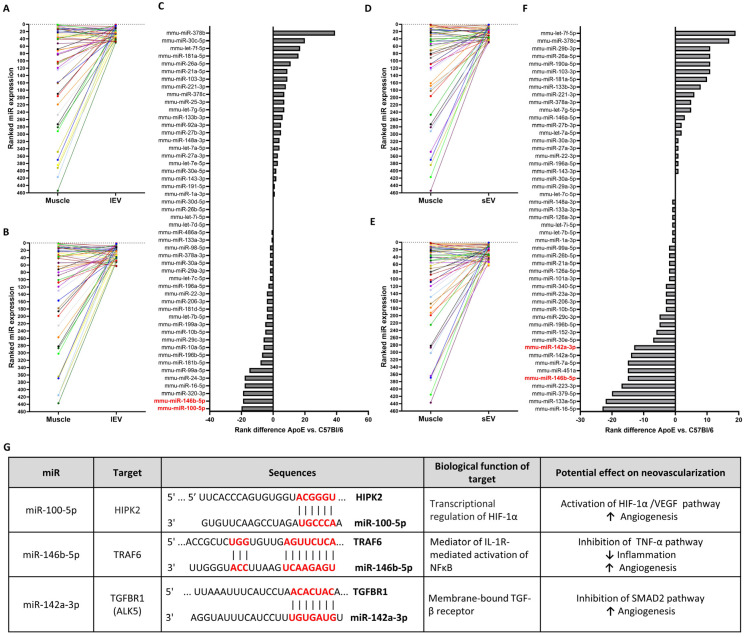
Effect of hypercholesterolemia on enrichment of miRs in EVs. (**A**,**B**). Leap in hierarchical ranking (expression ranking in lEVs vs. whole muscle) of 50 most expressed miRs in lEVs isolated from ischemic muscles of normocholesterolemic mice (**A**) or ApoE^−/−^ hypercholesterolemic mice (**B**). (**C**) Quantitative representation of effect of hypercholesterolemia on miR enrichment in lEVs. Potential angiomiRs identified are highlighted in red. (**D**,**E**). Leap in hierarchical ranking of 50 most expressed miRs in sEVs isolated from ischemic muscles of normocholesterolemic mice (**D**) or ApoE^−/−^ hypercholesterolemic mice (**E**). (**F**) Quantitative representation of effect of hypercholesterolemia on miR enrichment in sEVs. Potential angiomiRs identified are highlighted in red. (**G**) Description of targets, biological function, and angiogenic effects of angiomiRs, for which enrichment in EVs is impaired by hypercholesteremia. Binding sequences in target genes are shown in red.

The effect of HC on miR packaging in EVs within ischemic skeletal muscles is currently unknown. We tested the hypothesis that HC might impair the selective packaging of angiomiRs in EVs. Hierarchical ranking represents the relative expression of a given miRNA in a specific compartment (whole skeletal muscle or EVs) relative to other miRNAs within that compartment. An increase (or leap) in hierarchical ranking indicates that the miRNA is comparatively more enriched in EVs than in whole skeletal muscle. We first analyzed the leap in the hierarchical ranking of the 50 most expressed miRs by comparing their expression in whole ischemic skeletal muscles versus lEVs (Figure 4A) or sEVs (Figure 4D) within the ischemic muscles of control normocholesterolemic mice. We then investigated the effect of hypercholesterolemia on the enrichment of miRs in lEVs (Figure 4B) or sEVs (Figure 4E) in the ischemic muscles of hypercholesterolemic ApoE^−/−^ mice. Hypercholesterolemia was shown to impair the enrichment of several miRs in both lEVs (Figure 4C) and sEVs (Figure 4F).

Among the miRs that were the most affected by hypercholesterolemia, three were identified as potential angiomiRs within EVs (Figure 4G). The enrichment of miR-100-5p and miR-142a-3p is impaired in lEVs and sEVS, respectively, whereas miR-146b-5p is inhibited in both types of EVs. miR-100-5p targets the serine–threonine HIPK2, which can activate the PI3K/AKT and HIF-1a/VEGF pathways [33]. Small extracellular vesicle (sEV) miR-100-5p has also been shown to promote angiogenesis during the embryo implantation process [34]. miR-146b-5p has been shown to target TRAF6, thereby suppressing TNF-dependent inflammatory signaling and enhancing physiological angiogenesis [20]. In addition, miR-142a-3p targets the TGFBR1/ALK5 receptor, resulting in the inhibition of the SMAD2 pathway and subsequently promoting angiogenic responses [35].

### 2.5. Biological Pathways Predicted to Be Impaired by Hypercholesterolemia-Induced Alterations in lEV and sEV miRNA Cargo

In our study, we identified several candidate angiomiRs that are diminished by hypercholesterolemia in both lEVs and sEVs derived from ischemic muscle tissue (Figure 1, Figure 2, Figure 3 and Figure 4). We analyzed these miRs collectively and utilized miRPath v4 to predict their functional targets, as well as the pathway and gene unions influenced by this miR set.

**Figure 5:**
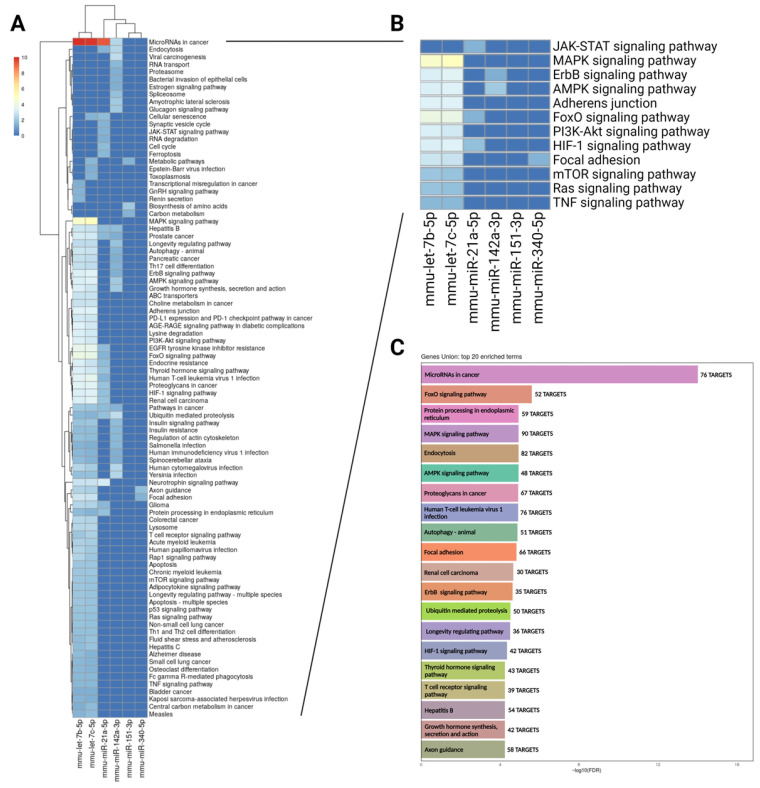
Predictive pathways modulated by miRs that are impaired by hypercholesterolemia in EVs within ischemic muscles. KEGG pathway unions (**A**,**B**) and gene unions (**C**) identified by miRPathv4 and predicted to be modulated by miRs that are impaired by hypercholesterolemia in lEVs and/or sEVs within ischemic muscles.

**Figure 6:**
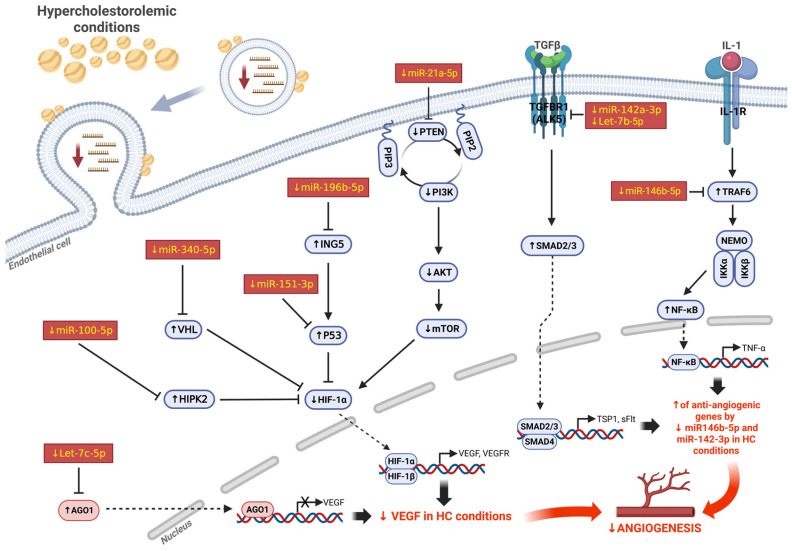
Integrated model of angiogenic effect of miRs that are impaired by hypercholesterolemia in EVs within ischemic muscles. Summary of potential role of EV-contained miRs that are impaired by hypercholesterolemia in ischemic muscles. Collectively, these miRs combine their physiological functions to promote angiogenesis and neovascularization through activation of multiple pathways.

As shown in Figure 5A,B, this set of miRs is collectively predicted to influence several major pathways implicated in angiogenesis, including HIF, MAPK, AMPK, Ras, and PI3K/Akt signaling. Gene union analysis (Figure 5C) further supports the fact that these miRs regulate numerous targets associated with angiogenesis-related processes, such as cancer pathways; focal adhesion; and MAPK, AMPK, and HIF-1 signaling. Taken together, our findings indicate that hypercholesterolemia alters the molecular cargo of both lEVs and sEVs in ischemic muscle, leading to reduced levels of specific miRs that jointly contribute to angiogenesis and ischemia-driven neovascularization through the activation of multiple pathways (Figure 6).

## 3. Discussion

To our knowledge, this is the first comprehensive study to characterize the impact of hypercholesterolemia (HC) on miR expression in EVs within ischemic skeletal muscles in the context of peripheral artery disease (PAD). It also represents the first comparison of miR cargo between EV subtypes—namely sEVs and lEVs—under conditions of HC and limb ischemia. While EVs are increasingly recognized as key regulators of physiological processes, their specific role in modulating neovascularization under pathological conditions remains poorly understood. To date, most studies have focused on EVs isolated from peripheral blood to identify miRs as potential biomarkers for atherosclerotic cardiovascular disease. Consequently, little is known about EVs derived from ischemic muscle tissue itself. In particular, how cardiovascular risk factors influence the miR content of these locally produced EVs—and how such changes may affect angiogenic responses—remains largely unexplored. Addressing this knowledge gap is critical, as EVs hold significant promise as therapeutic tools to enhance neovascularization and limit tissue damage in PAD.

Patients with atherosclerotic diseases often present several conditions that have been shown to impair neovascularization in response to ischemia [36]. This could explain the lack of efficacy of pro-angiogenic therapies in patients, compared to the positive results obtained in young and healthy animals [2,37]. HC is one of the most important conditions leading to the development of ischemic vascular diseases. First, it is involved in the initiation and growth of the atherosclerotic lesion, which progressively causes vascular obstruction, leading to acute or chronic tissue ischemia [38]. Second, hypercholesterolemia has also been associated with defective reparative processes occurring in response to ischemia. For example, reduced ischemia-induced neovascularization has been documented in several hypercholesterolemic animal models [11,12,13,39]. Moreover, the number and functional activity levels of PACs are reduced in hypercholesterolemic patients with or without clinically apparent atherosclerotic diseases [14,40]. However, the specific mechanisms that are involved in the inhibition of neovascularization and PAC function by hypercholesterolemia are not completely understood. The present study proposes a novel mechanism by which the modulation of miR expression in EVs within ischemic muscles could contribute to the impairment of ischemia-dependent reparative processes in hypercholesterolemic conditions.

A major mechanism by which EVs are believed to promote angiogenesis and neovascularization is their ability to deliver pro-angiogenic miRs (angiomiRs) to recipient cells [24]. In this study, we used a well-characterized mouse model of hindlimb ischemia [41] together with next-generation sequencing (NGS) to perform a comprehensive and unbiased assessment of miR expression profiles in ischemic skeletal muscles. To assess the impact of HC, we compared the results from hypercholesterolemic ApoE^−/−^ mice with those from normocholesterolemic control mice. Our results demonstrate that a few miRs are modulated by HC in whole ischemic muscles. Among the 50 most expressed miRs, we identified miR-151-3p as a potential angiomiR whose expression is significantly reduced by HC. miR-151-3p has been shown to enhance healing after spinal cord injury through the inhibition of p53 [42]. It has also been found to induce Slug-dependent angiogenesis in vitro [25]. miRs can function locally within the cells that produce them, but they are also critical mediators of paracrine signaling through their packaging into EVs. These EVs facilitate communication between cells by transferring bioactive molecules, including miRs. This mechanism is particularly relevant in the regulation of angiogenesis, where pro-angiogenic signals must be relayed to endothelial cells to promote new blood vessel formation. Therefore, analyzing the miR expression profile within EVs—rather than in whole skeletal muscle tissue—may offer more specific insights into the angiogenic signals being transmitted to endothelial cells in the local environment.

Interestingly, our NGS analysis indicates that the magnitude of miR modulation by HC is more pronounced in EVs compared to whole skeletal muscles (Figure 1, Figure 2 and Figure 3). We identified several miRs with potential pro-angiogenic effects (angiomiRs) that are reduced by HC in lEVs (Let-7b-5p, miR-151-3p, Let-7c-5p) or sEVs (miR-21a-5p, miR-196b-5p, miR-340-5p). Whereas miR-151-3p was also found to be reduced in the whole ischemic muscles of hypercholesterolemic ApoE^−/−^ mice, the other angiomiRs seem to be specifically inhibited in EVs. Let-7b-5p was previously identified in pericardial fluid sEVs and shown to be pro-angiogenic through targeting TGFBR1 in endothelial cells [26]. Let-7c-5p is another member of the Let-7 family involving hypoxia-responsive miRs that target argonaute 1 (AGO1), resulting in the translational desuppression of VEGF mRNA [27]. Let-7c-5p was also identified in sEVs, where it was shown to promote angiogenesis in multiple myeloma by polarizing M2 macrophages in the bone marrow microenvironment [28]. In a rat diabetic retinopathy model, miR-21a-5p was demonstrated to promote angiogenesis through the targeting of PTEN and activation of the PI3K/Akt/VEGF signaling pathway [29]. Interestingly, we previously showed that the level of miR-21a-5p is significantly increased by ischemia in sEVs [43]. miR-196b-5p targets ING5, which is involved in the activation of the PI3K/Akt pro-angiogenic pathway. Moreover, sEV-derived miR-196b-5p was previously shown to reduce CDKN1B, facilitating intercellular interaction and increasing angiogenesis in vitro [30]. miR-340-5p was shown to target von Hippel–Lindau (VHL) [31], a factor involved in the activation of the HIF-1a/VEGF pathway. Moreover, miR-340-5p in sEV was shown to promote angiogenesis in brain microvascular endothelial cells during oxygen glucose deprivation [32].

Our results also suggest that the packaging of specific miRs in EVs during ischemia might be impaired in hypercholesterolemic conditions. We found that the enrichment of miR-100-5p and miR-142a-3p is impaired in lEVs and sEVS, respectively, whereas the enrichment of miR-146b-5p is impaired in both types of EVs isolated from the ischemic muscles of hypercholesterolemic ApoE^−/−^ mice. miR-100-5p targets the serine–threonine HIPK2, which can activate the PI3K/AKT and HIF-1a/VEGF pathways [33]. During the embryo implantation process, miR-100-5p in sEVs was shown to promote angiogenesis [34]. We previously showed that miR-146b-5p inhibits TNF-dependent inflammation through the targeting of TRAF6, thereby increasing physiological angiogenesis [20]. Finally, miR-142a-3p was found to modulate VEGF and angiogenesis in vitro and in vivo [35,44]. Interestingly, we previously demonstrated that the level of miR-142a-3p is significantly increased by ischemia in sEVs [43].

Collectively our results suggest that in the setting of skeletal muscle ischemia, HC might impair the levels and selective packaging of specific angiomiRs in EVs. miR packaging within EVs is a complex process involving several miRNA-binding proteins such as heterogeneous nuclear ribonucleoproteins (e.g., hnRNPA2B1) and argonaute 2 (Ago2) that can bind specific miRs and shuttle them into EVs [45]. For larger vesicles (lEVs), membrane proteins including Caveolin-1 (Cav-1) are involved [46]. HC could alter membrane lipid composition, endosomal trafficking, and the function of RNA-sorting machinery, all of which are critical for EV biogenesis. These changes could in turn impair the selective packaging of microRNAs into EVs, leading to reduced or dysregulated miR export. However, the exact mechanisms through which HC disrupts the enrichment of angiomiRs within EVs in ischemic muscle remain unclear. Moreover, the functional impact of EV-packaged miRs—and how HC modifies their effects—is inherently complex, given that each miR can regulate multiple targets. In this study, we conducted bioinformatic analyses to predict functional miR targets and the physiological or pathological pathways potentially influenced by the miRs altered in EVs under HC conditions. Our findings indicate that, collectively, these miRs are predicted to affect several key angiogenesis-related processes, including cancer pathways; focal adhesion; and MAPK, AMPK, and HIF-1 signaling (Figure 5 and Figure 6).

## 4. Materials and Methods

### 4.1. Murine Hindlimb Ischemia

The protocol was approved by the Comité Institutionnel de Protection des Animaux (CIPA) of the Centre Hospitalier de l’Université de Montréal (CHUM). In this study, 6 to 8-week-old hypercholesterolemic ApoE^− t^ mice were purchased from Jackson Laboratory (Bar Harbor, ME, USA) and put on a Western-type diet (Teklad 88137, Envigo, Indianapolis, IN, USA) beginning 4 weeks before surgery and maintained until sacrifice [19]. C57BL/6 mice (Charles River, St-Constant, QC, Canada) were used as controls. The unilateral hindlimb ischemic model we used is the one originally described by Coufinhal et al. [12]. Briefly, the animals were anesthetized with 2% isoflurane, after which an incision was performed in the skin overlying the middle portion of the left hindlimb. After the ligation of the proximal end of the femoral artery, the distal portion of the saphenous artery was ligated, and the artery and all side branches were dissected free and excised. The skin was closed with a prolene monofilament (6-0) (Johnson & Johnson, Toronto, ON, Canada).

### 4.2. Isolation of Extracellular Vesicles

Two days after inducing ischemia, thigh and calf muscles from the ischemic limb were collected, rinsed in sterile D-PBS (Wisent, St-Jean-Baptiste, QC, Canada), and finely minced for 5 min in 37 mL of DMEM (Wisent). This time point was selected based on our earlier work showing that EV production in skeletal muscle increases markedly following ischemia, reaching a peak 2 days post-surgery [43]. The tissue suspension was first centrifuged at 3000× *g* for 3 min. The resulting supernatant was passed through a 70 µm cell strainer and centrifuged again at 3000× *g* for 3 min to eliminate remaining cells and debris. The clarified supernatant was then centrifuged at 20,500× *g* for 45 min to pellet lEVs, after which the remaining supernatant was ultracentrifuged at 120,000× *g* for 3 h to collect sEVs. Electron microscopy (Philips CM100 TEM, Amsterdan, The Netherlands) was used to characterize the isolated EVs including their size, morphology, and integrity of double-membrane vesicles (Supplemental Figure S1).

### 4.3. RNA Isolation and Next-Generation Sequencing Analyses

Total RNA was extracted from ischemic hindlimb muscles not depleted of EVs (i.e., whole muscles) using the Ambion mirVana miRNA isolation kit (Thermo Fisher Scientific, Carlsbad, CA, USA) according to the manufacturer’s protocol. Total RNA was extracted from EVs using the miRNeasy Mini Qiagen kit (Qiagen, Hilden, Germany). The quality of RNA was assessed with the Bioanalyzer RNA (Agilent, Santa Clara, CA, USA). Equal amounts of RNA samples (3–5/group) were pooled in each experimental condition: C57Bl/6 ischemic muscles (pool of 4), ApoE^−/−^ ischemic muscles (pool of 3), sEVs from C57Bl/6 ischemic muscles (pool of 4), sEVs from ApoE^−/−^ ischemic muscles (pool of 5), lEVs from C57Bl/6 ischemic muscles (pool of 3), and lEVs from ApoE^−/−^ ischemic muscles (pool of 3). Final RNA concentrations were then determined in each group, and an equal quantity of total RNA was used for library preparation. Library preparation was performed with the Truseq Small RNA library preparation kit (Illumina, San Diego, CA, USA) or Qiaseq miRNA library kit (Qiagen). Library quality was assessed with the Bioanalyzer DNA High Sensitivity (Agilent). Sequencing was performed on a NextSeq 500 (Illumina), yielding over 7 million reads per library. Normalization by library size was then performed and values expressed as reads per million reads mapped (RPM). We focused on miRs with an expression level of at least 100 RPM, and differential gene expression levels were expressed as the fold change between hypercholesterolemic conditions and normocholesterolemic controls. miRs were considered to be regulated if the fold change value was greater than 1.2 for upregulated miRs and lower than 0.8 for downregulated miRs. The data were deposited and are available on NCBI Gene Expression Omnibus (GEO), #GSE312883.

### 4.4. Culture of HUVECs

Human umbilical vein endothelial cells (HUVECs) were purchased from Thermo Fisher Scientific and cultured in medium 200 (Thermo Fisher Scientific) supplemented with low serum growth supplement (LSGS; 2% FBS, 3 ng/mL bFGF, 10 mg/mL heparin, 1 mg/mL hydrocortisone, and 10 ng/mL EGF; Thermo Fisher Scientific) and 100 IU/mL penicillin/0.1 mg/mL streptomycin (Wisent). Cells were grown at 37 °C, 5% CO_2_, and the medium was changed every 2 days. HUVECs were passaged when they reached 90% confluence, and passages 5–6 were used for all experiments.

### 4.5. miRNA Transfection in EVs

Transfection was performed to overexpress selected miRs in EVs isolated from the ischemic muscles of C57BL/6 mice. A commercially available miR-21a-5p mimic (hsa-miR-21a-5p) and let 7b-5p mimic (hsa-Let 7b-5p) were transfected in sEVs and lEVs, respectively (miRIDIAN Mimic; Dharmacon, Lafayette, CO, USA). A control miRNA mimic (mirCTL) was also transfected in both sEVs and lEVs (miRIDIAN Mimic Negative Control #1; Dharmacon). Transfections were carried out at a concentration of 120 nM of miR using the Exo-Fect siRNA/miRNA Transfection Kit (Systems biosciences, Palo Alto, CA, USA) according to the manufacturer’s protocol. Briefly, miR mimics were incubated with Exo-Fect siRNA/miRNA Transfection reagent for 15 min at room temperature. Then, isolated EVs were added to the mixture and incubated at 37 °C for 1 h. The mixture of EVs and miRs was then transferred to a pre-washed spin-column and incubated with gentle rotation for 15 min at room temperature. EVs were collected by centrifuging the spin-column for 30 s at 1000× *g*.

### 4.6. Capillary-like Structure Formation on Matrigel

The angiogenic activity of HUVECs was determined using a Matrigel capillary-like structure formation assay as previously described [47]. Briefly, HUVECs (20,000 cells/well) were either mixed or not with 10 μg/mL sEVs + miR-21a-5p mimic, sEVs + miRCTL, lEV + let 7b-5p mimic, or lEV + miRCTL in 100 μL M200 medium supplemented with 0.1% FBS. Then, cells were plated in 96-well plates precoated with 50 μL of growth factor-reduced Matrigel Matrix (Becton Dickinson Labware, Bedford, MA, USA) and cultured at 37 °C for 6 h. This was performed in duplicate for each treatment group. Capillary-like structures and junctions were observed and counted under a light microscope. Capillary-like structures were defined as a segment of elongated cells in a network, and junctions were defined as a point where capillary-like structures meet. Images were obtained at 50× magnification. n = 5/group for lEVs and 8/group for sEVs.

### 4.7. Bioinformatic Analysis

miR and target gene sequences were obtained from the miRDB [48,49] and TargetScan [50] databases. Pathway and gene union analyses were performed using miRPath v4 [51].

### 4.8. Statistical Analysis

The results for Matrigel assays are presented as the mean ± SEM. Statistical significance was evaluated by an ANOVA followed by a Bonferroni post hoc test. *p* < 0.05 was interpreted to denote statistical significance.

## 5. Conclusions

In a mouse model of peripheral artery disease, our study showed that HC is associated with a selective reduction in pro-angiogenic miRs (angiomiRs) in EVs produced locally within ischemic skeletal muscles. We observed that HC differentially affects miR expression in lEVs versus sEVs, yet collectively, the miRs diminished by HC were predicted to regulate several key pathways involved in angiogenesis and neovascularization. These findings represent an important initial step toward identifying angiomiRs that could be targeted to enhance the angiogenic function of EVs under hypercholesterolemic conditions. Looking forward, engineered EVs overexpressing specific angiomiRs may serve as effective angiogenic vectors to promote neovascularization and potentially reduce tissue damage in patients with HC and severe ischemic vascular disease.

## Data Availability

The original contributions presented in this study are included in this article. Further inquiries can be directed to the corresponding author.

## Supplementary Materials

The following supporting information can be downloaded at: https://www.mdpi.com/article/10.3390/ncrna12010003/s1, Figure S1: Transmission electron microscopy images of large (IEVs) and small extracellular vesicles (sEVs) isolated from ischemic skeletal muscle using differential centrifugation.

## Abbreviations

The following abbreviations are used in this manuscript:

PAD	Peripheral artery disease
HC	Hypercholesterolemia
EV	Extracellular vesicle
miRs	microRNAs
NGS	Next-generation sequencing
lEVs	Large EVs
sEVs	Small EVs
angiomiRs	miRs with potential pro-angiogenic effects
EC	Endothelial cell
VEGF	Vascular endothelial growth factor
HIF1	Hypoxia-inducible factor 1
PACs	Pro-angiogenic cells
HUVECs	Human umbilical vein endothelial cells
Ago1	Argonaute 1
VHL	Von Hippel–Lindau
Ago2	Argonaute 2
Cav-1	Caveolin-1
