# Supplementary figures and images for: Hypercholesterolemia Impairs the Expression of Angiogenic MicroRNAs in Extracellular Vesicles Within Ischemic Skeletal Muscles
Source: Noncoding RNA. 2026 Jan 26;12(1):3. doi: 10.3390/ncrna12010003 (PMC12922120; doi:10.3390/ncrna12010003)

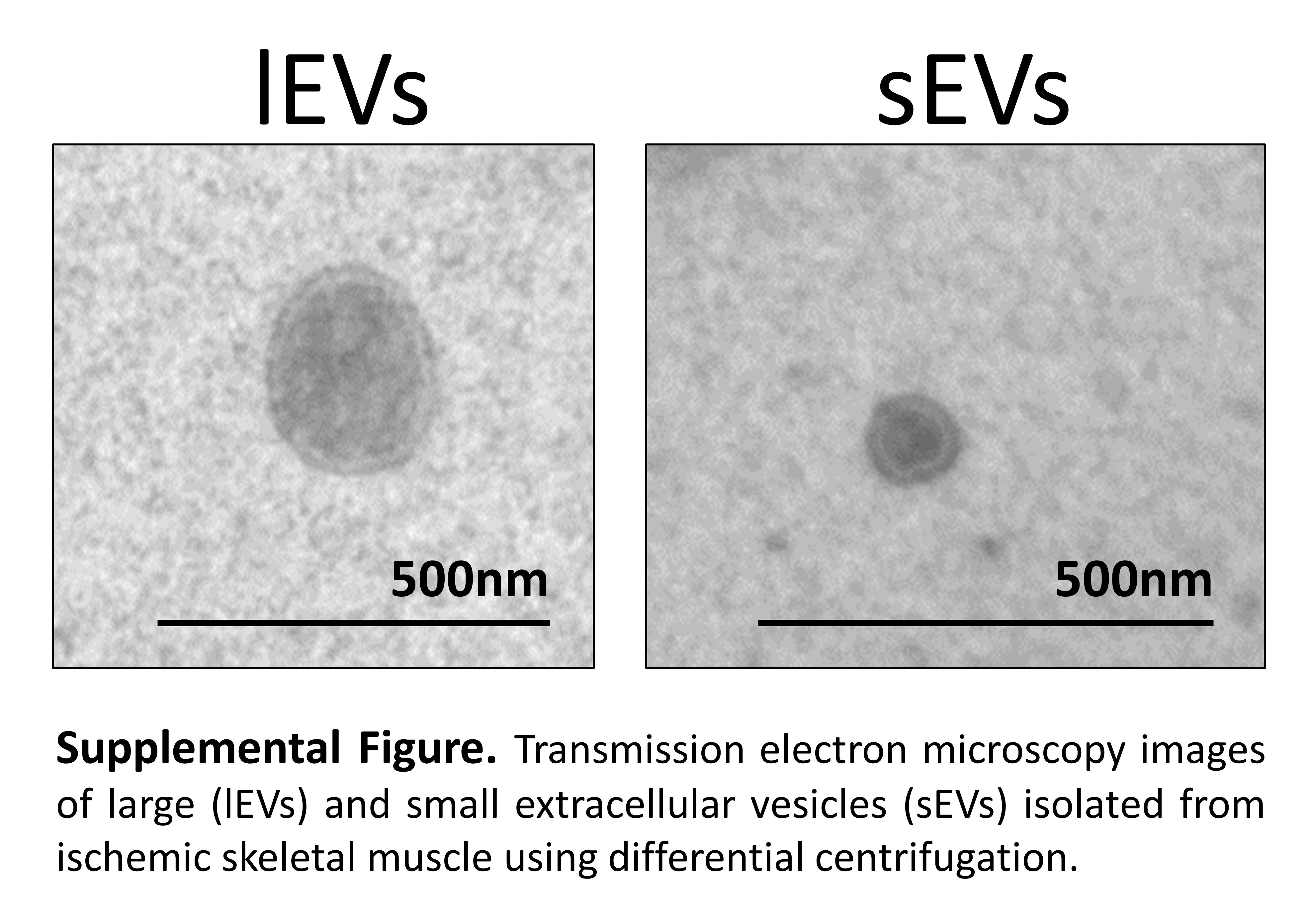

Supplement: Supplementary file 1 [file ncrna-12-00003-s001.zip › Figure S1.jpg]
